# MRI Evaluation of Anterolateral Ligament of the Knee: A Cross-Sectional Study in Malaysia

**DOI:** 10.7759/cureus.15758

**Published:** 2021-06-19

**Authors:** Ren Yi Kow, Chooi Leng Low, Khairul Nizam Siron@Baharom, Siti Nor Badriati Sheikh Said

**Affiliations:** 1 Department of Orthopaedics, Traumatology & Rehabilitation, International Islamic University Malaysia, Kuantan, MYS; 2 Department of Radiology, International Islamic University Malaysia, Kuantan, MYS; 3 Department of Radiology, Hospital Tengku Ampuan Afzan, Kuantan, MYS

**Keywords:** anterolateral ligament, knee, anterior cruciate ligament, magnetic resonance imaging, injury

## Abstract

Introduction

After detailed anatomical delineation of the anterolateral ligament (ALL) of the knee, there is a surge in research on this anatomical structure. Owing to the anatomical variation and lack of experience in the identification of this structure, magnetic resonance (MR) evaluation of the ALL produces mixed results. It was aimed to evaluate the ALL using the routinely performed MR imaging of the knee and to determine any associated factors with ALL injuries.

Materials and methods

Thirty-six MR images of the knee from 31 patients from January 1, 2017, to June 30, 2017, are evaluated. MR sequences performed include T1-weighted, T2-weighted, proton density (PD), and PD fat saturation (FS). All MR images were double-read by two authors and approved by a consultant radiologist with more than 20 years of radiological experience. The ALL was divided into three portions: femoral, meniscal, and tibial, and the ALL was considered fully visualized when all three portions were seen on MR images.

Results

At least a portion of the ALL was visualized in 27 scans (75%), and it was fully visualized in 20 scans (55.6%). The femoral portion was the most commonly identified (75%), followed by the meniscal portion (69.4%) and the tibial portion (58.3%). ALLs were best visualized on coronal view in PD FS with the lateral inferior genicular artery as a guide to locate the bifurcation of the meniscal and tibial components.

Conclusion

The ALL can be visualized in routine 1.5-T MR imaging, either full delineation (55.6%) or partially visualized (75%). It is best characterized via a PD-weighted sequence with fat saturation on the coronal plane. The ALL injury was associated with an anterior cruciate ligament (ACL) injury.

## Introduction

The anterolateral ligament (ALL) of the knee was described by Segond in 1897 as “a pearly, resistant fibrous band which shows tension during forced internal rotation” [1]. Previously, it was unclear whether the ALL is part of the iliotibial band or a separate ligament entity, as evidenced by various names it was called, including “short lateral ligament,” “capsule-osseous layers of iliotibial band,” “mid third lateral capsular ligament,” and “lateral capsular ligament” [2-5].

In 2013, Claes et al. delineate the ALL as a distinct ligamentous structure with consistent origin and insertion sites that subsequently renew the study interest on anterolateral soft tissue structures of the knee [6-8]. With a prevalence of 45% to 100% in cadaveric studies, ALL originates posterior and proximal to the lateral femoral epicondyle and inserts into the proximal tibia halfway between the fibula head and Gerdy’s tubercle, approximately 5 to 11 mm below the joint line [7,9]. The ALL also gives rise to a lateral meniscus insertion midway between the femoral origin and tibial insertion [7]. There are three components of ALL, namely, the femoral, meniscal, and tibial components [7].

Alongside anatomical studies, biomechanical studies also demonstrate that ALL is involved in the stabilization of knee rotation, especially during knee flexion of angles more than 30 degrees [10-15]. This is clinically significant, especially in patients with persistent anterolateral rotational instability of the knee as evidenced by a positive pivot shift test after anterior cruciate ligament (ACL) reconstruction [13].

Clinically, ALL reconstruction, in addition to ACL reconstruction, yields favorable outcomes. A meta-analysis by Kunza et al. shows that combined ACL and ALL reconstruction improves pivot shift and offers comparable clinical and functional outcomes compared with ACL reconstruction alone with no added complication [11]. A review by the multinational SANTI Study Group also demonstrates that the addition of ALL reconstruction to ACL reconstruction reduces the rate of ACL graft rupture, secondary meniscectomy, and instability, and improves the functional outcomes [10].

Previous studies in the identification of ALL with magnetic resonance imaging (MRI) revealed a mixed result. By utilizing a 3-Tesla (T) MRI, Hooda et al. managed to identify ALL in all 31 patients [16]. On the other hand, studies using 1.5T MRI show a large discrepancy, with reports showing full visualization of ALL ranging from 11% to 100%, possibly due to anatomical variations and the fine thickness of this structure [17-22]. Thus far, there is still no study on MRI evaluation of the ALL in South-East Asian countries. We aim to conduct a pilot study to evaluate the ALL of the knees using 1.5T MRI and to determine factors associated with ALL injuries.

## Materials and methods

This is a cross-sectional study to review MR images of knees performed in patients with suspected ligamentous injuries from January 1, 2017, to June 30, 2017. All the MRI knees are performed using a 1.5T MR scanner (Siemens Medical Solutions, MAGNETOM Aera, Malvern, PA) and Tx/Rx 15-channel knee coil (Siemans) with the following sequences: coronal proton density-weighted with fat saturation, T1 sagittal, sagittal proton density-weighted with fat saturation, T2 sagittal, and T2 axial (Table 1).

**Table 1:** Magnetic resonance imaging parameters used FS – fat saturation; Cor – coronal; PD – proton density; Sag – sagittal; TE – echo time (ms); TR – repetition time (ms); ETL – echo train length; BW – bandwidth (Hz); Freq – frequency (MHz); NEX – number of excitations; FOV – field of view (mm); Space – slice spacing (mm); Thick – thickness (mm)

Sequence	TE	TR	ETL	BW	Freq	Phase	NEX	FOV	Space	Thick
Cor PD FS	33	3200	5	159	63.64	100	1	160	0.3	3.0
Sag T1	12	600	Nil	151	63.64	100	1	190	0.4	4.0
Sag PD FS	49	4270	11	150	63.64	100	1	170	0.4	4.0
Sag T2	138	4400	12	120	63.64	100	2	140	0.4	4.0
Axial T2	96	3620	18	149	63.64	100	1	150	0.8	4.0

MR images of the following patients are excluded from this study: 1. Patients with a history of fractures or bony procedures; 2. Patients with a suspected tumor at the knee region; and 3. Patients having knee ligamentous surgeries in the past. All images are double-read by the lead two authors and are approved by a consultant radiologist with more than 20 years of radiology experience.

All components of the ALL (femoral, meniscal, and tibial) are assessed and are labeled as either visualized or not visualized. When all three components of the ALL are seen, it is classified as a fully visualized ALL. Next, the ALLs are assessed for any abnormality, as evidenced by any obvious discontinuity in the fibers, irregular contours associated with periligamentous edema, or proximal or distal avulsion, with or without associated bone fragment [22-23]. Other intra-articular structures of the knee, including ACL, posterior cruciate ligament (PCL), medial meniscus (MM), and lateral meniscus (LM), as well as the medial and lateral collateral ligaments (MCL and LCL), are assessed for any injury.

The findings of the ALL components are presented with a descriptive method. The association between ALL injuries and other intra-articular or extra-articular structural injuries is evaluated via Fisher’s Exact test.

## Results

A total of 36 knee MR images from 31 patients are included in this study period, with five patients being excluded (3 tumor cases and 2 postoperative scans). The demographic data is summarized in Table 2. More than 80% of the patients were of Malay ethnicity (n=27, 87.0%), followed by Indians (n=2, 6.5%) and Chinese (n=2, 6.5%). The majority of the patients included were males (n=22, 71.0%). The mean age of the patients is 29.4 years (range 13-51, SD 10.1). Slightly more than half of the scans are performed on the right knee (n=20, 55.6%). All patients had MRI of the knees to assess the ligamentous injury after pivoting injuries secondary to sports activities or falls.

**Table 2:** The demographic data of patients included in this study is demonstrated along with the breakdown of visualization of different components of the anterolateral ligament (ALL) SD - standard deviation

Demographic Factors		Number	Percentage
Gender	Male	22	71.0
	Female	9	29.0
Race	Malay	27	87.0
	Indian	2	6.5
	Chinese	2	6.5
Age		Mean 29.4	SD 10.1
Side	Left	16	44.4
	Right	20	55.6
Visualization of ALL (total 36 patients)	Femoral component	27	75
	Meniscus component	25	69.4
	Tibial Component	21	58.3
	Fully visualized	20	55.6

**Figure 1:**
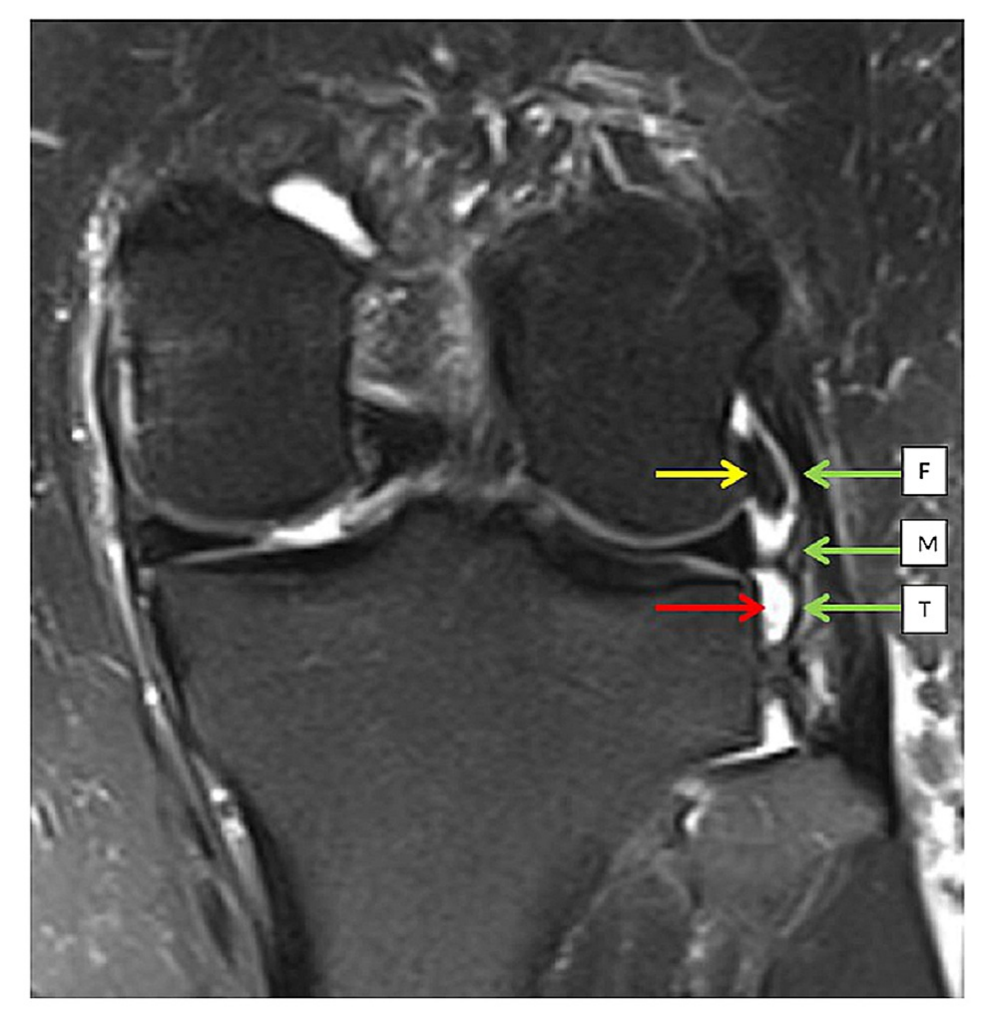
The anterolateral ligament (ALL) is best visualized on the PD-weighted fat saturation sequence on the coronal plane All three components of ALL (F: femoral, M: meniscal, and T: tibial) are visualized in this figure (green allow). The popliteus tendon is also visualized in this plane (yellow allow). The inferior lateral genicular artery (red arrow) can be used as a guide to identify the ALL.

We are able to delineate ALL fully in 20 scans (55.6%), and it is partly visualized (at least one part of the ALL was characterized) in 27 scans (75%). Out of the three components, the femoral part is the most commonly identified structure (n=27, 75%), followed by the meniscus part (n=25,69.4%) and the tibial part (n=21, 58.3%). ALLs are best visualized on PD FS coronal view, with the lateral inferior genicular artery acting as a guide to locate the bifurcation of the meniscal and tibial components (Figure 1). Delineation of ALLs from other surrounding structures, such as lateral collateral ligaments, iliotibial band, and popliteus tendon, is done in both coronal and axial views.

Out of the 36 knee MR images included in this study, 11 (30.6%) have ALL injuries. There are also 19 knees (52.8%) with evidence of ACL injuries. This is followed by injuries of the medial meniscus (n=18, 50%), lateral meniscus (n=16, 44.4%), PCL (n=3, 8.3%), and lateral collateral ligament (n=1, 2.8%). There is no medial collateral ligament injury identified among all the knee MR images in this series. Among the 19 knees with evidence of ACL injuries, nine of them have concomitant ALL injuries. The association of ALL injuries with injuries of other ligamentous structure are highlighted in Table 3. ACL injuries are found to be associated with ALL injuries (p=0.031). There is no association detected between ALL injuries and injuries of other structures such as medial meniscus (p=0.471), lateral meniscus (p=0.483), PCL (p=0.216), and lateral collateral ligament (p=1.0).

**Table 3:** The association between ALL injury and injury of other ligamentous structures is demonstrated All three components of ALL (F: femoral, M: meniscal, and T: tibial) are visualized in this figure (green allow). The popliteus tendon is also visualized in this plane (yellow allow). The inferior lateral genicular artery (red arrow) can be used as a guide to identify the ALL. There is no medial collateral ligament injury among the patients included. ACL – anterior cruciate ligament; PCL – posterior cruciate ligament; LM – lateral meniscus; MM – medial meniscus; LCL – lateral collateral ligament

Factors Associated With ALL injury		ALL injury		P-value	Odd Ratio
		No	Yes		
ACL injury	No	15	2	0.031	5.7
	Yes	10	9		
PCL injury	No	24	9	0.216	NA
	Yes	1	2		
LM injury	No	15	5	0.483	NA
	Yes	10	6		
MM injury	No	14	4	0.471	NA
	Yes	11	7		
LCL injury	No	24	11	1.000	NA
	Yes	1	0		

## Discussion

Paul Segond was the first to describe avulsion fracture of the proximal lateral tibia secondary to forced internal rotation of a flexed knee [1]. Multiple authors have hypothesized the anatomy and function of the ALL until Claes et al. characterize the anatomy of the ALL, which was inserted to the exact location described in the Segond fracture [1-6]. Although not fully understood, the ALL was hypothesized to be a lateral stabilizer, as evidenced by its role in controlling tibial rotation that affects the pivot shift phenomenon [6]. Injury of the ALL has been attributed to up to 10% of patients who have persistent rotational instability and pivot shift after a successful ACL reconstruction [13,17,24].

Despite detailed delineation of the ALL, MRI evaluation of the ALL remains challenging because of the anatomical variations and the fine thickness of this structure [6,24]. Aside from the high-resolution MR evaluation using a 3-T scanner, hitherto only one study demonstrates the identification of ALL in all the knee MR images [16,22]. Limited understanding of the detailed anatomy of the ALL, in addition to lower resolution and indecorous axis, may have contributed to the lower detection rate of the ALL [16]. Since most of the centers in Malaysia are performing MR imaging with 1.5-T scanners, the data from this study might serve as a guide for other centers to emulate.

Similar to the findings of Helito et al., we find that the ALL is best visualized on the coronal plane [18]. It should be noted that, due to the oblique course of the ALL through the anterolateral knee, it is difficult to characterize it in full (i.e, its entire path) within a single MR coronal slice [18]. Hence, non-visualization or partial visualization of the ALL may be attributed to an ALL injury or limitation of the MRI scan (thicker slice). To make the interpretation even more complicated, the tibial insertion of ALL may overlap with the articular capsule or iliotibial band, thus increasing the difficulty in isolating the ALL at the tibial insertion site [18]. We concur with this finding, as the tibial portion of the ALL is the least identified part in this study (58.3%).

For beginners to identify the ALL, we adopt the recommendations of Helito et al, in which the inferior lateral geniculate artery (ILGA), whenever it is present, is used as a landmark to identify the bifurcation of the meniscal and tibial portions of the ALL [18]. We find that the ILGA is present in 75% of the MR images. Upon identification of the ILGA and bifurcation of the ALL, the femoral and meniscal portions can be traced proximally and the tibial portion can be traced distally. For the proximal portion of ALL, we compared the axial view and the coronal view to delineate the ALL as the femoral epicondyle is a common origin of ALL, LCL, and popliteus muscle insertion.

In this series, a total of 11 scans (30.6%) reveal ALL injuries. As in our expectation, ALL injuries are associated with ACL injuries. This result mimics the one reported by Claes et al. and Ferretti et al., as there is an increased prevalence of ALL abnormalities in ACL-injured knees [25-26]. A validated computed model by Kang et al. shows that ALL is a secondary stabilizer to the ACL under gait and squat loading conditions in resisting anterior translation and internal rotation [27]. There is no association found between an ALL injury and injuries of other structures. This finding is consistent with the result reported by Helito et al. that there is no association between ALL injuries and meniscal injuries [28]. Nevertheless, Dyck et al. show that there is an association between ALL injuries and lateral meniscal injuries, owing to the anterolateral tibial displacement after an ALL injury with forces propagated to the lateral meniscus, causing tearing of the lateral meniscus [29]. These contrasting results cannot be verified with our case series due to the small sample size, hence future research with a larger sample size can be explored to fill up this knowledge gap.

Limitation

This study is limited by its small sample size and retrospectively collected data from a single institution, making it difficult to generalize the results to the whole population of Malaysia. As the MRI evaluation of ALL is at an inchoate stage, this study does not examine the inter-observer differences, as this pilot study is aimed to familiarise both the orthopedic surgeons and radiologists with the anatomy and technique to identify the ALL in the MR images. In this series, we examine MR images of both acute and chronic injuries, which might lower the detection rate of the ALL injury, as the ensuing fibrosis may mask the ALL injury [21]. Lastly, we did not include the patients’ clinical examinations (pivot test) for correlation with the MR findings. Nevertheless, this study provides a framework for a larger prospective multicentre study of the ALL, which incorporates both clinical and radiological findings of the ALL.

## Conclusions

The ALL can be visualized in routine 1.5-T MR imaging, either full delineation (55.6%) or partially visualized (75%). It is best characterized via PD-weighted and T2 sequences with fat saturation on the coronal plane. The most common identifiable part of the ALL is the femoral portion, whereas the least identified portion is the tibial part.
